# MEX3A knockdown inhibits the development of pancreatic ductal adenocarcinoma

**DOI:** 10.1186/s12935-020-1146-x

**Published:** 2020-02-28

**Authors:** Xing Wang, Yu-Qiang Shan, Qing-Quan Tan, Chun-Lu Tan, Hao Zhang, Jin-Heng Liu, Neng-Wen Ke, Yong-Hua Chen, Xu-Bao Liu

**Affiliations:** 1 Department of Pancreatic Surgery, West China Hospital, Sichuan University, No 37 Guo Xue Alley, Chengdu, 610041 Sichuan China; 2 Department of Hangzhou First People’s Hospital, No. 261, Huansha Road, Hangzhou, 310006 Zhejiang China

**Keywords:** PDA, MEX3A, Proliferation, Colony formation, Apoptosis, Migration

## Abstract

**Background:**

Pancreatic ductal adenocarcinoma (PDA) is one of the most serious causes of death in the world due to its high mortality and inefficacy treatments. MEX3A was first identified in nematodes and was associated with tumor formation and may promote cell proliferation and tumor metastasis. So far, nothing is known about the relationship between MEX3A and PDA.

**Methods:**

In this study, the expression level of MEX3A in PDA tissues was measured by immunohistochemistry. The qRT-PCR and western blot were used to identify the constructed MEX3A knockdown cell lines, which was further used to construct mouse xenotransplantation models. Cell proliferation, colony formation, cell apoptosis and migration were detected by MTT, colony formation, flow cytometry and Transwell.

**Results:**

This study showed that MEX3A expression is significantly upregulated in PDA and associated with tumor grade. Loss-of-function studies showed that downregulation of MEX3A could inhibit cell growth in vitro and in vivo. Moreover, it was demonstrated that knockdown of MEX3A in PDA cells promotes apoptosis by regulating apoptosis-related factors, and inhibits migration through influencing EMT. At the same time, the regulation of PDA progression by MEX3A involves changes in downstream signaling pathways including Akt, p-Akt, PIK3CA, CDK6 and MAPK9.

**Conclusions:**

We proposed that MEX3A is associated with the prognosis and progression of PDA,which can be used as a potential therapeutic target.

## Introduction

To date, pancreatic ductal adenocarcinoma (PDA) is the fourth most common cause of cancer-related deaths worldwide [1]. A large part of PDAC patients can only be diagnosed at an advanced stage and have non-specific symptoms before clinical manifestations. PDA mortality is almost equal to its morbidity and it is a fatal disease [2, 3]. After the intervention of surgery, chemotherapy and radiation therapy, the 5-year survival rate of pancreatic cancer is still less than 8% [4]. Moreover, distant metastases occur in 60% of patients within 24 months of surgery [5]. Albumin-bound paclitaxel (nab-paclitaxel) in combination with gemcitabine and fluorouracil is widely used as first-line treatment for metastatic PDA to improve survival and overall response rate [6–8]. Unfortunately, with the emergence of gemcitabine and fluorouracil resistance, the therapeutic efficacy of GEM in pancreatic cancer is declining [7, 8]. Recent research on PDA pointed out that the abnormal expression of genes or proteins plays an important role in the occurrence and development of this tumor [9, 10]. In general, it is urgent to deepen the understanding of the molecular mechanism of PDA and find new therapeutic targets.

MEX3 has a conserved region of 65 to 70 amino acids, including two K homology domains and one human gene family homologous to MEX3, MEX3A-D [11]. MEX3A is located in the paratope (156,072,013–156,081,998) and has 9986 base pairs [11]. The RNA-binding protein of evolutionarily conserved MEX3 family are first characterized in heterogeneous ribonucleoproteins, as mediators of post-transcriptional regulation in different organisms, participating in different physiological environments [12]. MEX3A has been identified to be associated with diseases, especially cancer, such as Wilms tumors [13], gastric carcinomas [14], colorectal carcinomas [15]. It is important to determine their effects on cancer development and assess their potential of cancer progression or prognosis.

## Materials and methods

### Cell culture

The PDA cell lines PANC-1 and SW1990 were cultured in 6-well plates with 5% CO_2_ in 37 °C humid air and supplemented with DMEM containing 10% fetal bovine serum (FBS). The medium was changed every 72 h, and subculture was performed with 0.05% trypsin and 0.02% EDTA at a concentration of 80%. After 24 h in DMEM without FBS, followup experiments were performed.

### Target gene RNA interferes with the preparation of lentiviral vector

MEX3A was used as a template to design multiple RNA interference target sequences, and the target sequence (5′-AGGCAAGGCTGCAAGATTAAG-3′) with the highest MEX3A knockdown efficiency was screened for downstream experiments. BRV-112 linear vector (Shanghai bioscienceres Co. Ltd., Shanghai, China) was obtained by restriction endonuclease Age I (NEB, # R3552L) and EcoR I (NEB, # R3101L) digestion. Construction of target gene RNA interference lentiviral vector. The products were transformed into the 100 µL TOP10 competent cells of *Escherichia coli* (TIANGEN, # CB104-03). After that, positive clones with correct sequencing were selected by PCR and then plasmids were extracted by using plasmid extraction kit (TIANGEN, # DP117). Virus packaging Helper plasmid (Helper 1.0, Helper 2.0) and the target plasmid were co-infected into 293T (lentivirus packaging cells). The supernatant of 293T cells was collected 48 h after infection for the quality test of lentivirus. Lentivirus vectors were labeled with fluorescence and observed under fluorescence microscope after infection 72 h (GFP, Cherry).

### qRT-PCR

Total RNA extraction according to sigma Trizol instructions (Invitrogen, Carlsbad, CA, USA). RNA reverse transcription was used to obtain cDNA using vazyme Hiscript QRT super mix (gDNA wiper) (Vazyme, Nanjing, China). The qRT-PCR was performed by using AceQ qPCR SYBR Green Master Mix (Vazyme, Nanjing, China). GAPDH was used as a reference control.

**Table:** 

Primer name	Sequence
MEX3A Primer-F	CGGAGTGGACTCTGGCTTTGAG
MEX3A Primer-R	CAGAGGAGAAGAGCACGGAGGT
GAPDH Primer-F	TGACTTCAACAGCGACACCCA
GAPDH Primer-R	CACCCTGTTGCTGTAGCCAAA

### Western blot analysis

The PDA cell lines PANC-1 and SW1990 were collected and lysed with RIPA lysis buffer (Cell Signal Technology, Danvers, MA, USA) according to the instructions. Quantitative extraction of proteins with BCA Protein Assay Kit (HyClone-Pierce, Waltham, MA, USA, # 23225). Then western blot analysis was performed by SDS-PAGE (10%). The protein was transferred to polyvinylidene fluoride (PVDF) membrane and incubated with 5% BSA containing 0.5% Tween 20 for 60 min, then incubated overnight at 4 °C with the following primary antibodies (see primary antibody information table for western blot). After washing with TBST, the blot was incubated with horseradish peroxidase (HRP) labeled polyclonal secondary antibody (1:3000) (Beyotime, Beijing, China, # A0208) at room temperature for 1 h. ECL and plus TM western blot system kit (Amersham, Chalfont, UK, # RPN2232) were used for color development.

**Table:** 

Name of antibody	Protein size (kDa)	Diluted multiples	Source of primary antibody	Company	Number
MEX3A	54	1:1000	Rabbit	Abcam	AB79046
N-cadherin	125	1:1000	Rabbit	Abcam	AB18203
Vimentin	54	1:2000	Rabbit	Abcam	AB92547
Snail	29	1:1000	Rabbit	Abcam	3879S
Akt	60	1:1000	Rabbit	CST	4685
p-Akt	60	1:1000	Rabbit	Bioss	BS5193R
CDK6	37	1:1000	Rabbit	Abcam	AB15127
PIK3CA	110	1:1000	Rabbit	Abcam	AB40776
MAPK9	48	1:1000	Rabbit	Abcam	AB76125
GAPDH	37	1:3000	Rabbit	Bioworld	AP0063

### MTT assay

First, PANC-1 and SW1990 cells were trypsinized, completely suspended and counted. The cell density was 2000 cells/well and inoculated to 96-well plates (100 µL/well) (Corning, Corning, NT, USA, # 3599) overnight, and the cells were repeated 3–5 times in each group. 5 mg/mL MTT (5 mg/mL) (3-(4, 5-dimethylthiazol-2-yl)-2, 5-diphenyl tetrazolium bromide) (Genview, Beijing, China; # JT343) 20 μL was added 4 h before the end of the culture from the day after the planking, without changing the fluid. After 4 h, the medium was completely removed, 100 µL DMSO was added. The oscillator was oscillated for 5 min, OD value was detected by the enzyme-connected immunodetector 490/570 nm and the data were recorded for analysis.

### Colony formation assay

PANC-1 and SW1990 cells were trypsinized, and the culture medium was completely suspended to prepare a cell suspension. The cells cultured in the 6-well plate, 1000 cells/well were inoculated for 8 days, medium was changed every 3 days, and cell status was observed. Cell clones were photographed under fluorescence microscope before the termination of the experiment, and the cells were washed with PBS. 1 mL 4% paraformaldehyde was added to each well, the cells were fixed for 50 min and washed with PBS. After that, GIEMSA dye solution was added to each well for 20 min, dried them, cell clones were then photographed for counting.

### Apoptotic assay

PANC-1 and SW1990 cells were cultured with 6-well plates, 2 ml/well, digested with trypsin, and then the cell suspension was suspended, centrifuged at 1300 rmp for 5 min, and supernatant was discarded. Cell precipitate was washed with 4 °C pre-cooled D-Hanks (pH = 7.2–7.4). The cells were washed with 1 × binding buffer for precipitation, and centrifuged at 1300 rmp for 3 min to collect the cells. 200 μL 1 × binding buffer was added to suspend cell precipitation, followed by 10 μL Annexin V-APC staining at room temperature and dark for 15 min. Finally, 1 × binding buffer 500 μL was added and tested on machine.

### Transwell assay

100 μL serum-free medium was added and placed in the incubator for 1–2 h. PANC-1 and SW1990 cells were digested with trypsin, and the cell suspension was prepared by resuspension with low serum medium. Carefully removed the medium from the small chamber and added 600 μL containing 30% FBS to the lower chamber. The cells were inoculated in 24 well plates 100,000 cells/well, 100 μL/well in inner chamber and 600 μL/well in outer chamber for 24 h. Place the chamber upside down on the blotting paper to remove the medium and gently remove the metastatic cells using a cotton swab. Added 400 μL stain to the hole in the 24-well plate and soak the chamber in the staining solution for 20 min, dyed the cells on the lower surface of the membrane to transfer the cells. Soaked the chamber in a large water cup and rinsed it in the air after washing it several times. Microscope photo membrane dissolved at 10% acetic acid, detection of absorbent 0D540.

### Human apoptosis antibody array

The intracellular cell signaling pathway was examined using the Human Apoptosis Antibody Array Kit (# AB134001). All in all, the PANC-1 cells were collected after lentivirus infection for 3 days, washed with PBS, lysed with lysis buffer 2–8 °C for 30 min, and then gently shaken. The total extracted protein was diluted with the array diluent buffer kit to 0.5 mg/mL. Each array antibody membrane was blocked with blocking buffer for 30 min at room temperature, which incubated at 4 °C and gently shaken overnight. 1 × Biotin-conjugated Anti-cytokine was incubated overnight at 4 °C and gently shaken. HRP linked Streptavidin was added to the membranes. Protein was visualized using ChemiDoc XRS chemiluminescence detection and imaging system. The density of the spots was quantitated using Quantity One software and normalized to the α–tubulin levels.

### Animal xenograft model

Animal research was approved by the ethics committee of West China Hospital, Sichuan University conducted in accordance with guidelines and protocols for animal care and protection. BALB/c female nude mice (4 weeks old) were purchased from Shanghai Jiesijie Experimental Animals Co., Ltd (Shanghai, China). PANC-1 cells with luciferase reporter tag infected with shMEX3A or shCtrl were subcutaneously injected into BALB female nude mice (5 × 10^6^ cell per mouse). Data were collected (the weight and volume of the tumor) after 19 days of injection of PANC-1 cells, and then measured per week up to 45 days. Subsequently, D-luciferin (15 mg/mL) was injected into the mouse peritoneum at a dose of 10 μL/g, waiting for about 15 min, and then placing it in a dark room for bioluminescence imaging. The tumor load was evaluated weekly with bioluminescence imaging, and the IVIS spectral imaging system (emission wavelength 510 nm) analyzed. 10 min before in vivo imaging, anesthesia was performed by inhaling with 3% isoflurane. After 45 days, the mice were executed, injected with sodium pentobarbital, removed from the tumor and taken a photo, weighing.

### Immunohistochemical staining

63 cases of survival-time PDA and matched normal adjacent cancer tissue were purchased from Shanghai Outdo Biotech Co., Ltd (Shanghai, China). The informed consents were collected from tissue donors (patients). Specimens were fixed in formalin and embedded in paraffin (FFPE). Xylene were used for paraffin section dewaxing 15 min per time and 100% alcohol for hydration 10 min. After repairing and blocking of the citrate antigen, the sample and MEX3A antibody (1: 1000, Abcam, USA, # AB79046) were incubated overnight in an incubator at 4 °C. After elution with PBS for 5 times, secondary antibody IgG (1: 400, Abcam, USA, # AB6721) was added, incubated at room temperature for 30 min, and washed with PBS for 3 times. Tissue slices were first stained with DAB, and then with hematoxylin. Finally, images were taken under a microscope and evaluated according to the German immune response score [16]. In summary, the high or low expression level of MEX3A in PDA tissues is defined by the median based on total score of positive cells and total staining intensity.

### Ki67 staining

Tumor tissue was sectioned from the sacrificed mice. After repairing and blocking of the citrate antigen, antibody Ki67 (1: 200, Abcam, USA, # AB16667) was added to the shMEX3A or shCtrl, respectively. After mixing, incubated overnight at 4 °C. PBS elution for several times, IgG (1: 400, Abcam, USA, # AB6721), secondary antibody was added and incubated at room temperature for 30 min. PBS was washed again. Tissue slices were first stained with DAB, and then with hematoxylin. Images were collected with a photomicroscope and analyzed.

### Statistical analysis

The qRT-PCR was analyzed by 2 ^−∆∆CT^ method. *T* test were used to compare the difference. P values less than 0.05 were considered statistically significant. The data are expressed as mean ± SD (n ≥ 3) and analyzed using GraphPad Prism 6 software (GraphPad Software Inc., San Diego, CA, USA).

## Results

### Upregulation of MEX3A in PDA tissues

**Fig. 1:**
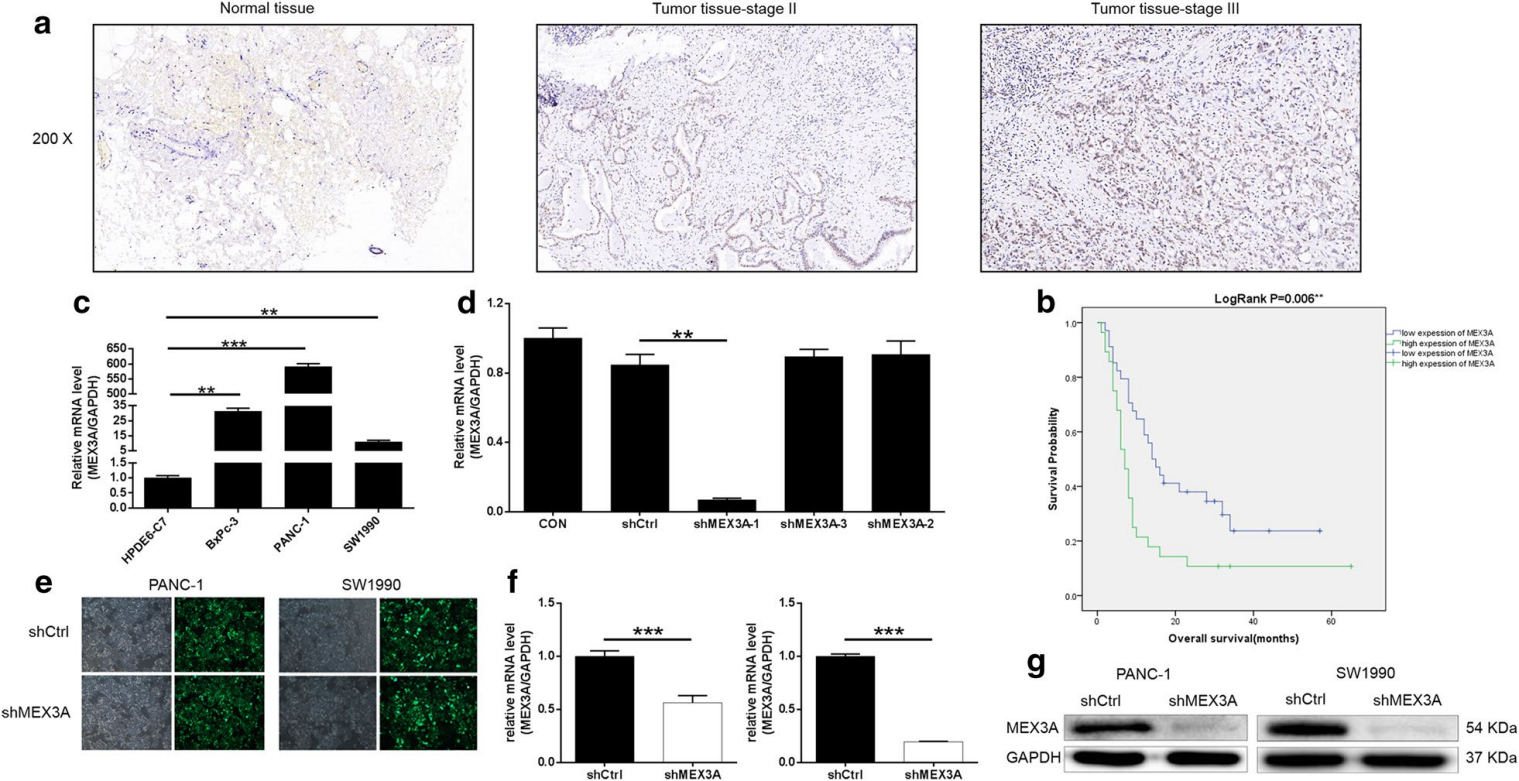
MEX3A is highly expressed in PDA and the construction of MEX3A knockdown cell model. **a** Expression levels of MEX3A in PDA tumor tissues and adjacent normal skin tissues were detected by IHC staining. **b** Kaplan–Meier survival analysis MEX3A expression and overall survival of PDA. **c** MEX3A expression in HPDE6-C7, BxPc-3, PANC-1 and SW1990 cells was detected by qRT-PCR. **d** qRT-PCR was used to screen knockdown efficiency of MEX3A in shMEX3A-1, shMEX3A-2, and shMEX3A-3 groups. **e** Infection efficiency for PANC-1 and SW1990 cells was evaluated by expression of green fluorescent protein 72 h post-infection. **f**, **g** The specificity and validity of the lentivirus-mediated shRNA knockdown of MEX3A expression was verified by qRT-PCR (**f**) and western blot analysis (**g**). The data were presented as the mean ± SD (n = 3). *P < 0.05, **P < 0.01, ***P < 0.001

According to the Immunohistochemical (IHC) staining (Fig. 1a and Table 1), the expression of MEX3A in PDA tissues was significantly higher than that in normal tissues (*P *<0.001), which allowed the subsequent correlation analysis between MEX3A expression and clinicopathological data. Further, according to Mann–Whitney U analysis (Table 2), we revealed that there was a significant association between the expression of MEX3A and pathological grade. Moreover, the similar results were also displayed by Spearman rank correlation analysis (Table 3). Based on the Kaplan–Meier survival analysis (Fig. 1b) we showed that the expression of MEX3A was significantly correlated with the overall survival of PDA patients. In conclusion, MEX3A might be associated with the development and prognosis of PDA.

**Table 1:** Expression patterns in pancreatic cancer tissues and normal tissues revealed in immunohistochemistry analysis

MEX3A expression	Tumor tissue		Normal tissue		P value
	Cases	Percentage (%)	Cases	Percentage (%)	
Low	34	54.8	52	98.1	< 0.001
High	28	45.2	1	1.9

**Table 2:** Relationship between MEX3A expression and tumor characteristics in patients with pancreatic cancer

Features	No. of patients	MEX3A expression		P value
		Low	High	
All patients	62	34	28	
Age (years)				0.462
< 67	30	15	15	
≥ 67	32	19	13	
Gender				0.704
Male	36	19	17	
Female	26	15	11	
Lymph node number				0.302
≤ 6	30	23	7	
> 6	24	7	17	
Lymph node positive				0.687
≤ 0	34	18	16	
> 0	24	14	10	
Tumor size				0.467
< 4 cm	23	14	9	
≥ 4 cm	39	20	19	
Grade				0.023
I	1	0	1	
II	32	23	9	
III	29	11	18	
AJCC Stage				0.782
1	5	3	2	
2	44	26	18	
4	4	2	2	
T stage				0.936
T1	2	1	1	
T2	7	4	3	
T3	37	21	16	
N stage				0.593
N0	34	18	16	
N1	25	15	10

**Table 3:** Relationship between MEX3A expression and tumor characteristics in patients with pancreatic cancer

	Pearson correlation	MEX3A
Grade	Pearson correlation	0.292
	Significance (double tailed)	0.021
	N	62

### Construction of MEX3A knockdown cells models

As shown in Fig. 1c, qRT-PCR indicated that the expression of MEX3A in cells BxPc-3, PANC-1, and SW1990 was relatively high compared to HPDE6-C7 cells (*P *< 0.05). Moreover, Fig. 1d showed that the shMEX3A-1 group has the highest knockdown efficiency of MEX3A, reaching 91.9% (*P* < 0.01) for downstream experiments. PANC-1 and SW1990 cells were infected with shMEX3A for silencing MEX3A, while that infected with shCtrl were used as negative control. Fluorescence imaging (Fig. 1e) was made 72 h after infection of PANC-1 and SW1990 with shMEX3A or shCtrl, and the results showed that the efficiency of cell infection reached over 80% and the cell state was normal. Results of qRT-PCR (Fig. 1f) displayed that the knockdown efficiencies of MEX3A in PANC-1 and SW1990 cells were 43.8% and 80.5%, compared with the shCtrl groups, respectively. Western blot (Fig. 1g) results showed that the expression of MEX3A protein in the shMEX3A group was downregulated after lentivirus infection compared with the shCtrl group. Our data suggested that MEX3A knockdown cell models were successfully constructed.

### Knockdown of MEX3A inhibited PDA cells proliferation and colony formation

**Fig. 2:**
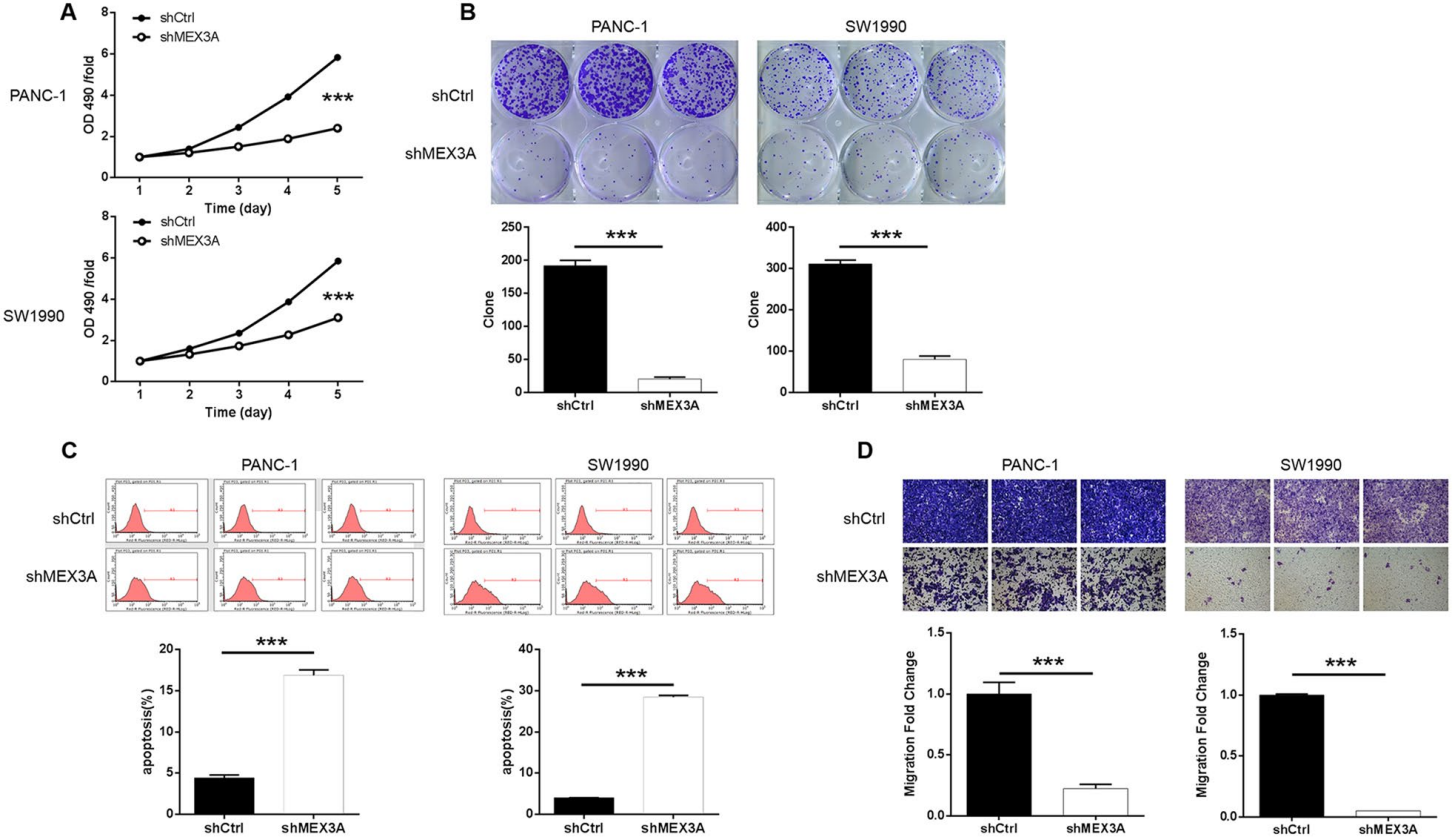
Knockdown of MEX3A inhibits cell proliferation and migration, promotes apoptosis in PDA cells. **a** Cell proliferation of PANC-1 and SW1990 cells with or without knockdown of MEX3A was evaluated by MTT assay. **b** Colony formation was evaluated for PANC-1 and SW1990 cells with or without MEX3A knockdown. **c** Flow cytometry analysis based on Annexin V-APC staining was utilized to detect the percentage of early apoptotic cell for PANC-1 and SW1990 cells. **d** Cell migration of PANC-1 and SW1990 cells with or without knockdown of MEX3A was evaluated by Transwell assay. The data were expressed as mean ± SD (n = 3), *P < 0.05, **P < 0.01, ***P < 0.001

Subsequently, MTT assay and colony formation assay were performed to detected cell proliferation and colony formation. First, MTT assay (Fig. 2a) suggested that the proliferation of the PANC-1 and SW1990 cells in the shMEX3A group decreased (*P* < 0.001). Colony formation capability is another character for malignant tumors. The effect of MEX3A knockdown on the colony formation of PANC-1 and SW1990 cells were observed by Giemsa staining, which indicated that the number of colonies in the shMEX3A group was significantly less than that in the shCtrl group (*P* < 0.001) (Fig. 2b). These experimental results suggested that MEX3A may play an important role in the cell proliferation of PDA.

### Knockdown of MEX3A promoted PDA cells apoptosis

To further investigate the role of MEX3A in the development of PDA, flow cytometry was applied to evaluate the percentage of apoptotic cells among the cells infected with shMEX3A or shCtrl (indicated by Y-axis: the green fluorescence from GFP on lentivirus). Compared with shCtrl group, MEX3A downregulation significantly promoted the apoptosis of PANC-1 and SW1990 cells. The apoptosis rates in PANC-1 cells increased by 12.47%, while that in SW1990 cells increased by 24.45% (P< 0.001) (Fig. 2c). It is easy to conclude from our results that the downregulation of MEX3A significantly promotes the apoptosis of PDA in PANC-1 and SW1990 cells.

### Knockdown of MEX3A inhibited PDA cells migration

In order to investigate the role of MEX3A in the metastasis of PDA, its effects on cell migration ability was measured by Transwell assay. The knockdown of MEX3A significantly inhibited cell migration in PANC-1 and SW1990 cells in comparison to the shCtrl groups. The migration rate of in PANC-1 and SW1990 cells increased by about 78% and 95%, respectively (*P *< 0.001) (Fig. 2d). These results lead to the conclusion that MEX3A promoted cell migration in PANC-1 and SW1990 cells of PDA.

### Exploration of downstream molecular mechanism of MEX3A in PDA cells

**Fig. 3:**
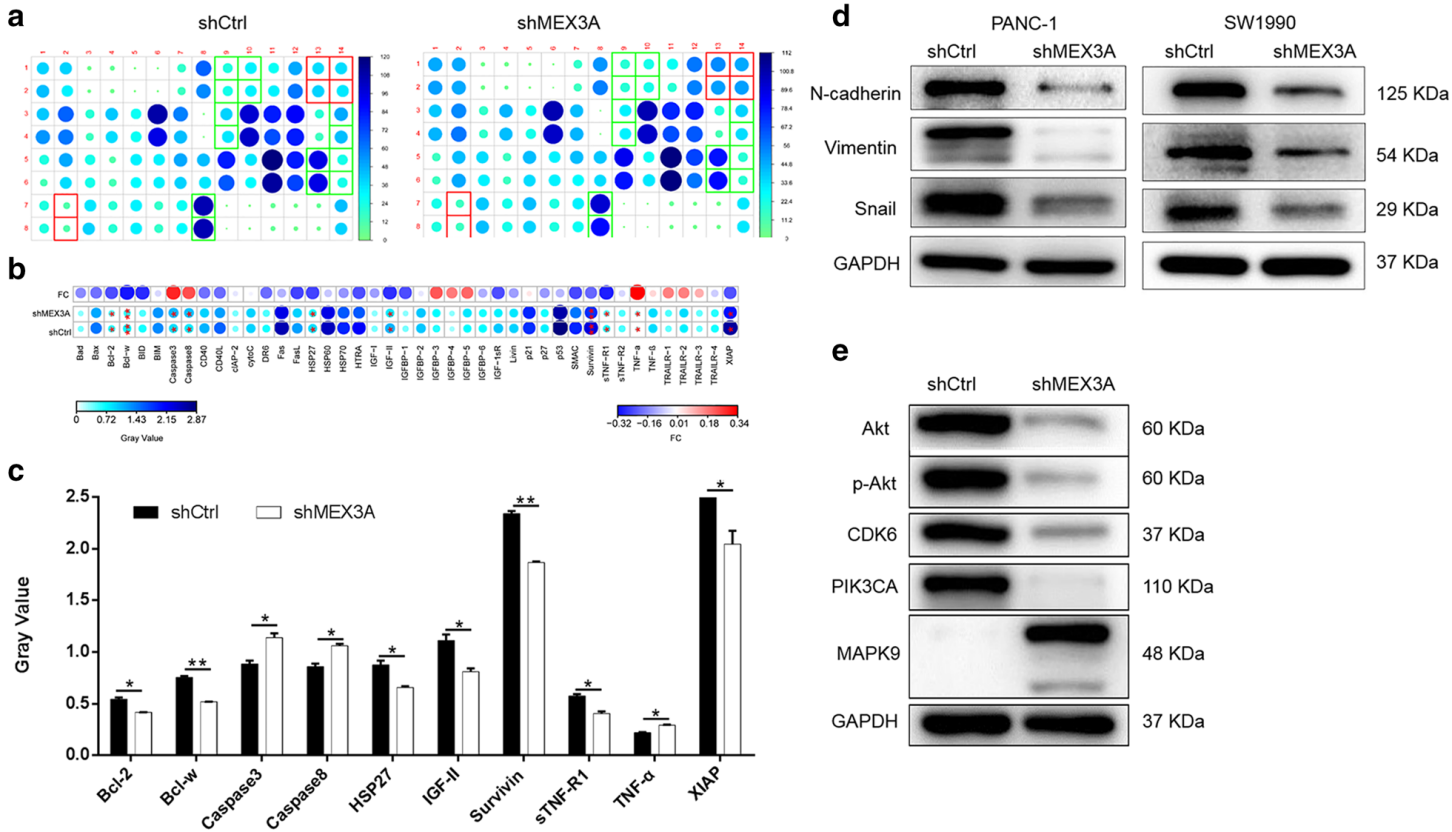
Exploration of downstream molecular mechanism of MEX3A in PDA cells. **a** Human apoptosis antibody array analysis was performed in PANC-1 cells with or without MEX3A knockdown. **b** Differences in human apoptotic antibody array were analyzed in PANC-1 cells regardless of MEX3A knockdown. **c** Densitometry analysis was performed and the gray values of differentially expressed proteins were shown. **d** The expression of epithelial–mesenchymal transition (EMT) proteins were observed by western blot in PANC-1 and SW1990cells. **e** The expression of target protein pathway was observed by western blot in PANC-1. The data were expressed as mean ± SD (n = 3), *P < 0.05, **P < 0.01, ***P < 0.001

For exploring the potential mechanism of the regulation ability of MEX3A knockdown in PDA, human apoptosis antibody array was performed to analyze the differential expression of 43 proteins in PANC-1 cells between shMEX3A and shCtrl groups. As shown in Fig. 3a–c, among the tested proteins, the expression levels of pro-apoptotic proteins including Caspase3, Caspase8 and TNF-α were significantly upregulated while the expression levels of protein Bcl-2, Bcl-w, HSP27, IGF-II, Survivin, sTNF-R1 and XIAP of anti-apoptotic proteins were significantly downregulated in shMEX3A group.

Moreover, the expression of epithelial–mesenchymal transition (EMT) proteins were observed with western blot, such as N-cadherin, Vimentin and zinc finger-related transcription factor (Snail) and so on, to explore the mechanism of these proteins in the development of PDA. Western blot (Fig. 3d) showed that the expression of N-cadherin, Vimentin and Snail in the PANC-1 and SW1990 cells were downregulated in shMEX3A compared with the shCtrl group. In addition, compared with shCtrl, the expression of Akt, p-Akt, PIK3CA, and CDK6 were downregulated, while the expression level of MAPK9 was upregulated in MEX3A knockdown PANC-1 cells detected by western blot (Fig. 3e). These results were consistent with the aforementioned cellular experiments especially the cell apoptosis assay.

### Knockdown of MEX3A in PDA cells impaired tumorigenesis in vivo

**Fig. 4:**
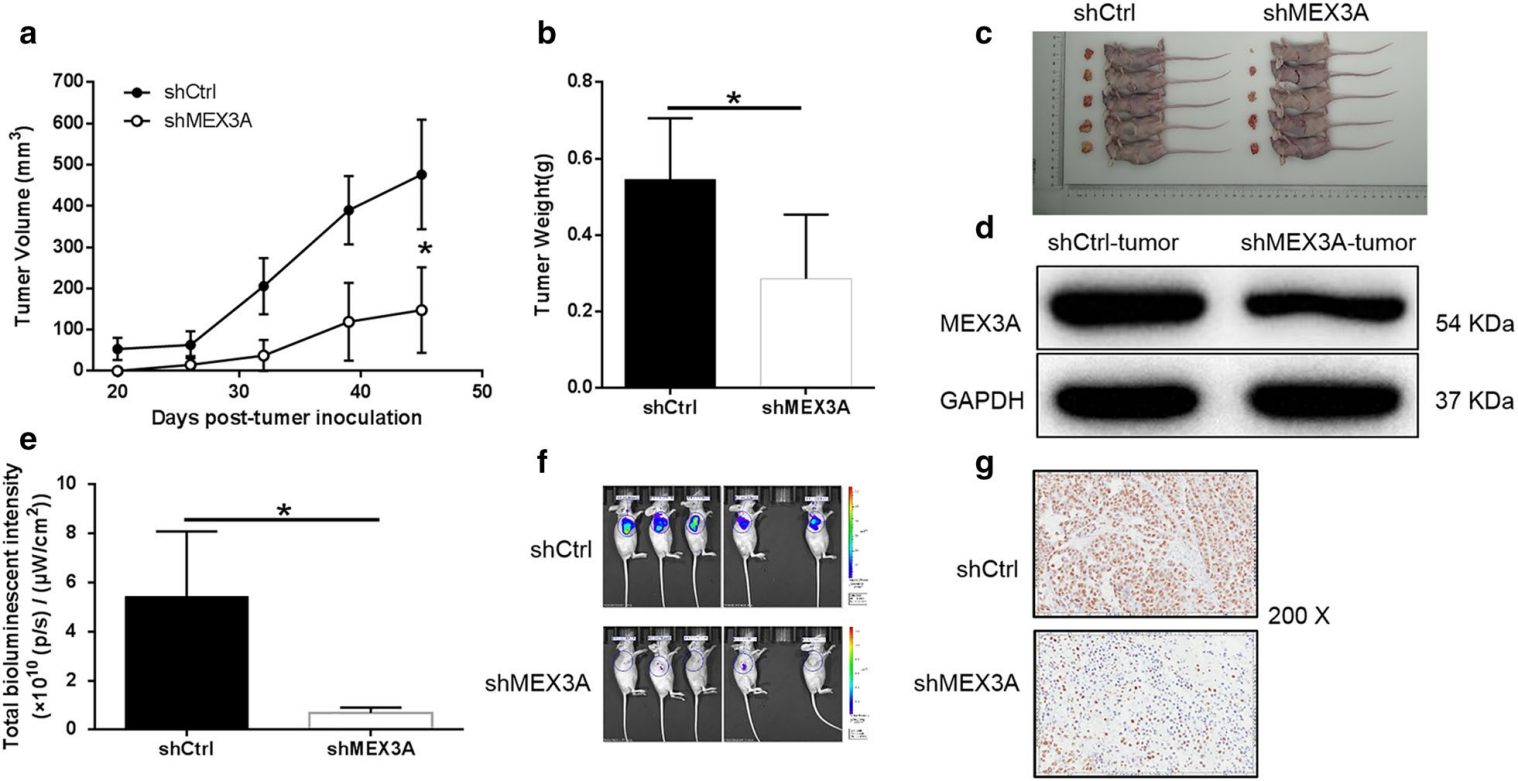
Knockdown of MEX3A inhibits tumor growth in mice xenograft models. **a** The volume of tumors in shCtrl group and shMEX3A group was measured after post-injection. **b** The average weight of tumors in shCtrl group and shMEX3A group. **c** Images of mice and tumors in shCtrl group and shMEX3A group. **d** Detection of MEX3A expression in tumors of mice models of shCtrl and shMEX3A groups by western blot. **e** The total bioluminescent intensity of tumors in shCtrl group and shMEX3A group. **f** The bioluminescence imaging of tumors in shCtrl group and shMEX3A group. **g** The Ki67 staining of tumor tissues in shCtrl group and shMEX3A group. The data were expressed as mean ± SD (n = 3), *P < 0.05, **P < 0.01, ***P < 0.001

The above studies confirmed that downregulation of MEX3A could inhibit cell proliferation, migration and promote apoptosis in vitro. We still want to explore whether the knockdown of MEX3A has consistent results in vivo. Therefore, PANC-1 cells with or without MEX3A knockdown were subcutaneously injected into nude mice to establish mouse xenotransplantation model. The tumor volume in the shMEX3A group was obviously smaller than that in the shCtrl group (*P* < 0.05) (Fig. 4a). The average weight of tumors in mice inoculated shMEX3A cells was 0.386 ± 0.118 g, which was significantly lower than that of the shCtrl group (*P* < 0.05) (Fig. 4b, c). In addition, from the western blot results, compared with tumors of shCtrl group, the protein MEX3A expression of shMEX3A group tumor was downregulated (Fig. 4d).

Additionally, bioluminescence imaging suggested that tumor growth in shMEX3A group was slower than that in shCtrl group (P < 0.05) (Fig. 4e, f). The results of Ki67 staining showed that the proliferation index of tumor tissues in shMEX3A group was significantly lower than that in the shCtrl group (Fig. 4g). In summary, the results of in vivo experiments confirmed the correctness of the conclusions of in vitro experiments, indicating that MEX3A played a regulatory role and impaired tumorigenicity in PDA.

## Discussion

MEX3A plays various roles in biological processes. Baumgart et al. pointed out that the expression of MEX3A is similar to that of mitotic markers of proliferating cell nuclear antigen [17]. To date, few studies have evaluated the effects of MEX3A on tumor cells. For example, MEX3A overexpression is associated with the recurrence of Wilms tumors [13]. In addition, Jiang et al. confirmed that abnormal activation of MEX3A in human gastric cancer cells promoted cell proliferation and migration [14].

Chiaravalli et al. suggested that a decrease in Mex3A expression may result in more pronounced inhibition of bladder cancer cell growth [18]. Interestingly, Adiseshaiah et al. found that MEX3A expression was not a poor prognostic factor for bladder urothelial carcinoma [19]. Importantly, MEX3A expression is involved in the regeneration of all intestinal epithelial cells with slow kinetics, helping to maintain cell renewal during chemotherapy and radiation therapy [20]. Based on this, we are urged to explore the regulation mechanism of MEX3A in PDA and provide feasible treatment strategies for PDA.

In this study, it was found that MEX3A is not only highly expressed in tumor tissues and human cultured cells of PDA, but also significantly correlated with patients’ prognosis. In addition, MEX3A knockdown inhibits PDA cell proliferation, migration, promotes apoptosis, and disrupts the cell cycle. Specifically, in vivo studies demonstrated that decreased tumorigenicity after knockdown of MEX3A, which is consistent with in vitro studies.

Escape from apoptosis is the basis of cancer pathogenesis [21]. Apoptosis involves a series of biochemical events, which are mediated by a variety of cellular signals. Moreover, MEX3A knockdown promotes PDA cell apoptosis through a series of apoptosis-related proteins, such as upregulating pro-apoptotic proteins, including Caspase3, Caspase8, and TNF-α; while downregulating anti-apoptotic proteins, such as Bcl-2, Bcl-w, HSP27, IGF-II, Survivin, sTNF-R1 and XIAP. It has been known previously that binding of TNF to TNF-R1 initiates the Caspase activation pathway through the intermediate membrane protein TNF receptor-related death domain (TRADD) and death domain protein (FADD) [22]. The Bcl-2 family is a key regulator for supporting and resisting apoptosis, and perturbations may indicate cell-to-cell death or irreversibility [23]. Furthermore, Schafer et al. proposed that HSP27 is involved in the regulation of PDA apoptosis pathway [24]. Recently, Momeny et al. clarified that Cediranib inhibits the proliferation of PDAC cells by inhibiting the anti-apoptotic proteins Survivin and XIAP and induces apoptosis [25]. In view of the above, it can be presented that MEX3A knockdown to promote PDA cell apoptosis is a complex process of apoptotic protein regulation.

This study found that MEX3A knockdown inhibited PDA cell migration while down-regulating EMT markers such as N-cadherin, Snail and Vimentin. EMT is a developmental process in which cells acquire the ability to migrate [26]. EMT is essential for the vigorous movement of cells during embryogenesis, and tumor cells can reactivate the EMT program and increase their aggressiveness [26]. David et al. Point out that carcinogenicity associated with EMT is a feature that must be selected during cancer progression [27]. Momeny et al. found that cediranib can attenuate the migration and invasion of PDA cells by reducing the expression of EMT markers ZEB1, N-cadherin and Snail [25]. The vitamin D analog MART-10 inhibits metastatic potential by downregulating EMT in PDA cells [28]. Accordingly, it is possible to support PDA cell migration through EMT.

Furthermore, the knockdown of MEX3A affects various activities of PDA cells and involves the expression of downstream signal pathways Akt, p-Akt, PIK3CA, CDK6 and MAPK9. It has been reported that PI3K/Akt/NF-κB/mTOR is the main signal transduction axis that controls cell proliferation, survival, apoptosis and malignant transformation [29]. PIK3CA mutations can cause pancreatic tumorigenesis and can be targeted by PI3K inhibitors proposed by Payne et al. [30]. Deeb et al. demonstrated that inhibiting survival (anti-apoptosis) Akt/NF-κB/mTOR signal transduction affects PDA cell proliferation, cycle arrest and apoptosis [31]. In addition, Liu et al. proposed that anti-CDK4/6 treatment can induce EMT and enhance PDA cell invasion by activating SMAD-dependent TGF-b signaling [32]. Moreover, a large amount of evidence showed that MAPK9 signaling is related to lung cancer, breast cancer, colon cancer, and ovarian cancer, mainly to adenocarcinoma cells [33]. From these reports, it is inferred that the occurrence of various activities of PDA cells is jointly regulated by a complex network system.

All in all, it was concluded that MEX3A was involved in the development and progression of PDA and that it could be potential prognosis indicator and therapeutic target. However, this study still has shortcomings. For example, the number of specimens included in this study is limited, and the underlying mechanism of MEX3A-mediated PDA regulation remains unclear. Therefore, in the future work, we will further deepen the understanding of the molecular mechanism of PDA related to MEX3A.

## Data Availability

The datasets used and/or analyzed during the current study are available from the corresponding author on reasonable request.
